# A selective pretreatment method for determination of endogenous active brassinosteroids in plant tissues: double layered solid phase extraction combined with boronate affinity polymer monolith microextraction

**DOI:** 10.1186/1746-4811-9-13

**Published:** 2013-04-18

**Authors:** Jun Ding, Li-Jing Mao, Bi-Feng Yuan, Yu-Qi Feng

**Affiliations:** 1 Key Laboratory of Analytical Chemistry for Biology and Medicine (Ministry of Education), Department of Chemistry, Wuhan University, Wuhan, 430072, China

**Keywords:** Brassinosteroid, Boronate affinity chromatography, Polymer monolith microextraction, Double layered solid phase extraction, HPLC-ESI-MS/MS

## Abstract

**Background:**

Brassinosteriods (BRs), a group of important phytohormones, have various effects on plant growth and development. However, their physiological functions in plants have not been fully understood to date. Endogenous BRs in plant tissue are extremely low and the elucidation of BRs functions relies on sensitive detection method. Reported methods for the determination of BRs required large amount of plant tissue, tedious pretreatment process, and were lack of selectivity. Therefore, development of a simple and selective method for the sensitive quantification of BRs is highly needed.

**Results:**

We established a pretreatment method of BRs in plant tissues by employing double layered solid phase extraction (DL/SPE) combined with boronate affinity polymer monolith microextraction (BA/PMME). After the initial depigmentation with DL/SPE cartridge, BA/PMME was employed to selectively extract BRs from sample matrix. Uniquely, most sample matrix was successfully removed by BA monolith purification. Using this method, BRs was determined by liquid chromatography-mass spectrometry (LC-MS). Endogenous active BRs could be detected in only 1 g fresh weigh (FW) leaves or 0.5 g FW flower tissues.

**Conclusion:**

A DL/SPE-BA/PMME pretreatment method for the determination of endogenous brassinosteroids in plant tissues was developed and validated. The proposed method was sensitive and selective. Besides, it may be further developed for the determination of other BRs including their precursors and conjugates.

## Background

Brassinosterods (BRs), confirmed as the sixth plant hormone, are a group of naturally occurring polyhydroxy steroids [1]. Since the first BR was discovered in 1970 [2], around 60 natural occurrence of BRs have been reported with wide occurrence in the plant kingdom [1]. BRs have multiple functions on various physiological and metabolic processes and normally occur in extremely low concentration. Many biologists have been dedicated to the researches on signal transduction, biosynthesis, degradation and metabolic pathway of BRs [3-5]. The studies of BRs functions rely on the availability of selective and sensitive method for the quantification of endogenous BRs in plant tissues.

As for phytohormone analysis, liquid chromatography-electrospray ionization-tandem mass spectrometry (LC-ESI-MS/MS) is a dominant analytical technique due to their high selectivity and sensitivity [6-8]. However, the concentrations of endogenous BRs in plant tissue are extremely low, and matrix of plant extracts are complicated. Therefore, direct analysis of BRs in plant matrix by LC-ESI-MS/MS is unpractical. To circumvent this problem, it is necessary to develop an effective pretreatment method to eliminate the interference of the sample matrix. Up to date, liquid-liquid extraction (LLE), solid phase extraction (SPE), magnetic solid phase extraction (MSPE), solid phase microextraction (SPME), HPLC purification or their combinations were extensively employed in BR pretreatment [9-13]. However, most of the previously reported methods were tedious, solvent-consuming, and required large amount of plant materials (more than 5 g fresh weight (FW)). Besides, BRs were normally isolated from plant extract based on hydrophobic or hydrophilic interaction; thus it was inevitable that some other similar components would be co-extracted with BRs, which burdens the following liquid chromatographic separation and suppresses the response signal of target analytes during mass spectrometry analysis. Therefore, it’s essential to develop a selective and sensitive sample pretreatment method.

**Figure 1:**
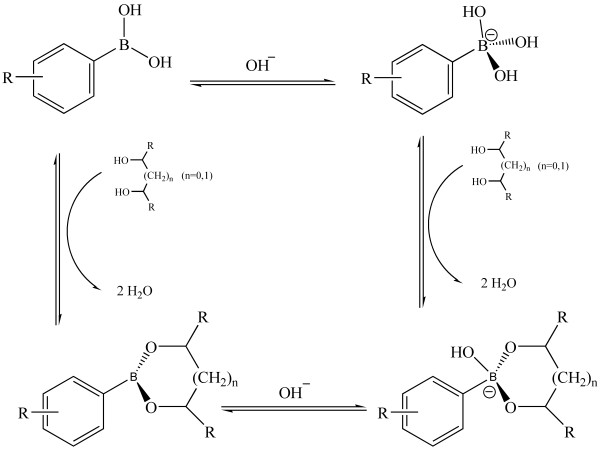
Mechanism of boronic affinity reaction.

**Figure 2:**
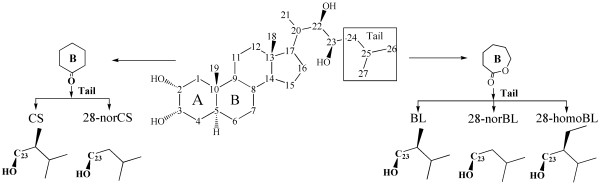
Structure of five endogenous BRs (CS, 28-norCS, BL, 28-norBL, 28-homoBL).

Boronate affinity chromatography is a powerful tool for the selective separation and enrichment of cis-diol-vcontaining compounds from sample matrix [14,15]. The mechanism relies on the reversible pH controlled formation/dissociation of cyclic esters. Under alkaline conditions, boronic acids can form five or six membered cyclic esters with cis-diols, while the esters decompose once in acidic solution (Figure 1). This capture/release principle has drawn much attention in sample pretreatment filed. Boronate affinity magnetic nanoparticles, bonded silica and polymer monoliths have been prepared for the capture and separation of catechol, carbonhydrates, RNA, nucleosides, glycoproteins and glycopeptides [15-21]. As BRs possess two pairs of cis-diols at C_2_, C_3_ and C_22_, C_23_ position (Figure 2), boronate affinity chromatography could be a powerful technique for the selective isolation of BRs from plant matrix. Takatsu and co-workers carried out the pioneering work of BRs analysis using boronate affinity chromatography, in which phenylboronic acid (PBA) gel was used as extraction medium [22]. In their method, sample was loaded in dispersive mode, which required pyridine as catalyst and heating for several hours. Then the BRs-adsorbed sorbents were packed in a SPE cartridge followed by desorption. The whole procedure was tedious and time-consuming. Whereas, polymer monolith microextraction (PMME), one kind of solid phase microextraction using polymer monolith as the extraction material, has several merits such as less consumption of solvent and sample, fast mass transfer and low backpressure in applications due to a binary porous structure (mesopores and macropores) of the polymer monoliths [23-27]. In our previous research, a boronate affinity monolith, poly(3-acrylamidophenyl boronic acid-co-ethylene dimethacrylate)(AAPBA-*co*-EDMA) was successfully prepared by a one-step in situ polymerization procedure. The prepared poly(AAPBA-*co*-EDMA) shows excellent performance for the selective isolation of glycopeptides and glycoproteins by PMME [28]. Due to the convenient synthesis process of the monolith and easy operation of the PMME process, the boronate affinity monolith is expected to be an appropriate tool for the extraction of BRs.

**Figure 3:**
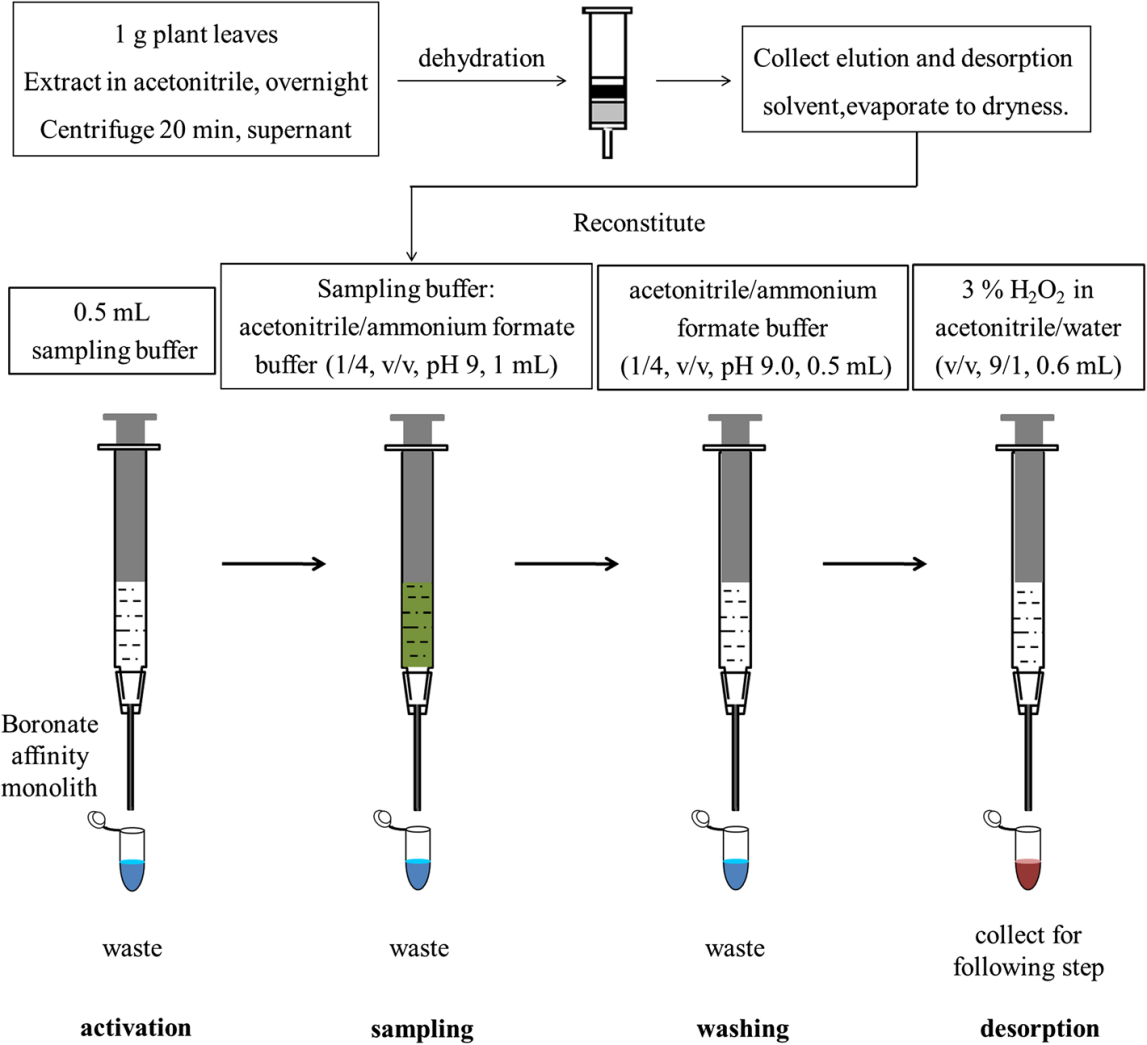
Sample pre-treatment protocol for brassinosteroids.

In this study, a selective pretreatment method was developed for the determination of endogenous active BRs in plant tissue by combing double layered solid phase extraction with boronate affinity polymer monolith microextraction (Figure 3). The whole pretreatment process involved an initial purification with a double layer SPE (GCB/PSA) cartridge [29] followed by the selective extraction of BRs with BA monolith. With our method, endogenous BRs can be detected in 1 g (FW) leaves or 0.5 g (FW) flowers by LC-MS.

## Results and discussion

Five active forms of BRs (BL, CS, 28-norBL, 28-homoBL, 28-norCS) were chosen as targets to evaluate the performance of our analytical method. Among them, BL, CS and 28-norCS represented the most important BRs because of their wide distribution as well as their potential biological activity [1].

### Optimization of PMME conditions

BA/PMME is the core step in the whole sample preparation procedure. In order to obtain high extraction efficiency and good purity of BRs in plant matrix, conditions of PMME process were optimized, including pH of sampling solution, acetonitrile content in sampling solution, sampling rate, type of desorption solvent, acetonitrile content in desorption solvent and desorption rate.

**Figure 4:**
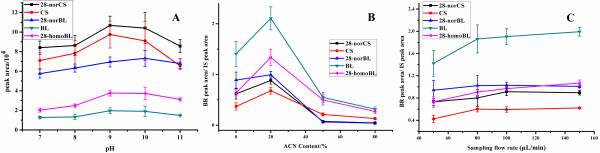
**Optimization of sampling conditions.** (**A**) Effect of pH on the BR extraction efficiencies. (**B**) Effect of acetonitrile content in sampling solution on the BR extraction efficiencies. (**C**) Effect of sampling flow rate on the BR extraction efficiencies. The sampling solution was spiked with five BRs standard solution (4 ng for each BR).

As boronate affinity chromatography is pH-dependent, pH of sampling solution can affect the extraction efficiencies of BRs. Optimization of pH was carried out in phosphate buffer in the pH range of 7–11. As shown in Figure 4A, extraction efficiencies of BRs increased from pH 7 to pH 10, and then dropped at pH 11. The reaction efficiencies between BRs and boronic acid were low when the pH was below 8; and the structure of BRs may be damaged when pH was over 11. To achieve the highest extraction efficiencies, pH 9.0 was selected for further study.

BRs are hydrophobic molecules with polar groups; thus acetonitrile content in sampling solution would affect the solubility of BRs in aqueous buffer, while high content of acetonitrile might affect the reaction efficiency of BRs with boronate affinity monolith. Therefore, acetonitrile content in sampling buffer was investigated in the range of 0-80% (v/v). As shown in Figure 4B, the extraction efficiencies increased from 0 to 20%, and then dropped with the increase of acetonitrile content from 20% to 80%, which suggested that with 20% acetonitrile content in the sampling solution, BRs were endowed with fair solubility and the boronic acid-BR reaction efficiencies were acceptable. So 20% acetonitrile was adopted in the following experiments.

Since the boronate affinity extraction was accomplished through covalent bond, the reaction efficiency might be related to the sampling flow rate. The flow rate was investigated in the range of 50–150 μL/min. The result indicated that the sampling flow rate has minor influence on the extraction efficiencies (Figure 4C), and higher flow rate may cause the damage of the column due to the high pressure. Therefore, the flow rate of 100 μL/min was selected.

**Figure 5:**
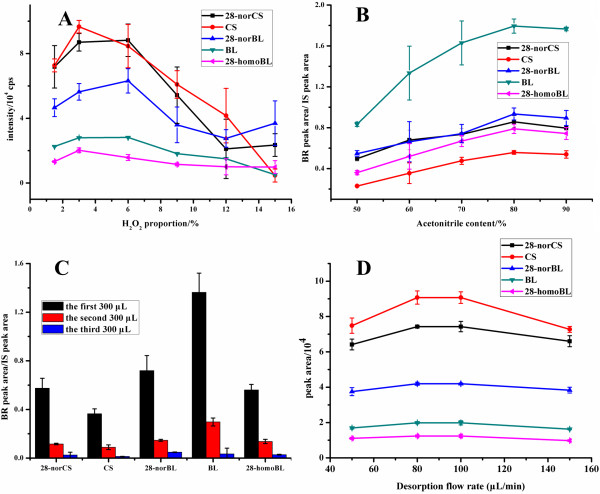
**Optimization of desorption conditions.** (**A**) Effect of H_2_O_2_ proportion in desorption solution on the BR desorption efficiencies. (**B**) Effect of acetonitrile proportion in desorption solution on the BR desorption efficiencies. (**C**) Effect of desorption volume on the BR desorption efficiencies. (**D**) Effect of desorption flow rate on the BR desorption efficiencies. The sampling solution was spiked with five BRs standard solution (4 ng for each BR).

BRs possess a pair of cis-diols, which are able to form five-membered boron-oxygen heterocyclic ring on the monolith in the PMME loading process. To desorb BRs from the monolith, various desorption solutions were investigated, including formic acid, phosphoric acid and trifluoroacetic acid. However, the heterocyclic ring at C22, C23 position is too stable to dissociate under acidic condition due to its large steric hindrance [10,30]. Fortunately, H_2_O_2_ was previously reported as an effective reagent to split C-B bond and then release diol-containing substances [31]. Accordingly, H_2_O_2_ content in desorption solvent was investigated within the range of 1.5% -15% (H_2_O_2_ proportion in acetonitrile/water (v/v, 1/1), v/v). It was found that relatively good desorption efficiencies could be obtained with 3% H_2_O_2_ (Figure 5A). However, high content of H_2_O_2_ in desorption solution caused the decrease of desorption efficiencies, which might be attributed to the damage of BRs under high content of H_2_O_2_. Therefore, 3% H_2_O_2_/acetonitrile solution was adopted. In addition, BRs could be adsorbed onto the monolith due to hydrophobic interaction; thus it was necessary to investigate acetonitrile content in desorption solvent and the volume of desorption solvent. As shown in Figure 5B, the desorption efficiency increased with the increase of acetonitrile content; and 0.6 mL desorption solution containing 90% acetonitrile was efficient to elute out the BRs (Figure 5C). Therefore, 0.6 mL of acetonitrile/water (v/v, 9/1) with 3% H_2_O_2_ (v/v,) was used as the desorption solution.

The effect of flow rate on desorption efficiency was also investigated in the range of 50–150 μL/min (Figure 5D). No remarkable effect of the flow rate was observed onto the desorption efficiencies. And the desorption rate of 100 μL/min was selected.

Taken together, sample was dissolved in acetonitrile/buffer (1/4, v/v, pH 9.0, 1 mL), and loaded onto the boronate affinity monolith at the flow rate of 100 μL/min. After washing with the washing solution of acetonitrile/buffer (1/4, v/v, pH 9.0, 0.5 mL), the extracted BRs were desorbed with 3% H_2_O_2_ in acetonitrile/water (9/1, v/v, 0.6 mL) at the flow rate of 100 μL/min.

### Comparison of two pretreatment methods

**Figure 6:**
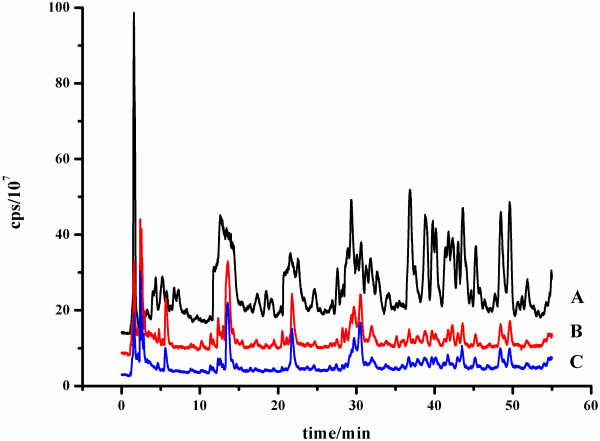
**Comparison of extraction performance between the methods of DL/SPE-LLE and DL/SPE-BA/PMME.** (**A**) *O. sativa* leave samples (1 g FW spiked with five BRs at 2 ng for each BR) treated with DL/SPE-LLE method; (**B**) *O. sativa* leave samples (1 g FW spiked with five BRs at 2 ng for each BR) treated with DL/SPE-BA/PMME method. (**C**) Standard sample (1 mL sampling solution spiked with 5 BRs at 2 ng for each BR) extracted with boronate affinity monolith.

Recently we developed DL/SPE-LLE (double layered SPE-liquid liquid extraction) method for the extraction of BRs from plant samples [29]. The DL/SPE-LLE method was convenient, but the selectivity was not satisfactory. To solve this problem, the boronate affinity extraction was applied for the further purification of BRs from the double layered SPE (DL/SPE) in our current study. And the performance of these two methods (DL/SPE-LLE and DL/SPE-BA/PMME) was compared (Figure 6). Figure 6A and 6B are the TIC chromatograms of plant sample (spiked with BR standards) treated with DL/SPE-LLE and DL/SPE-BA/PMME method, respectively. And Figure 6C represents TIC chromatogram of BR standards dissolved in acetonitrile/buffer (1/4, v/v, pH 9.0) treated with BA/PMME. It can be seen that the purification performance of DL/SPE-BA/PMME method (Figure 6B) is much better than that of DL/SPE-LLE method (Figure 6A). And it is similar to the BR standards without plant matrix (Figure 6C). The results suggest the high selectivity of our newly developed method. In addition, it should be mentioned that PMME require much smaller amount of solvent compared with the liquid-liquid extraction.

### Method validation

The calibration curve was constructed by plotting the mean peak area ratio of analytes to internal standard ([^2^H_3_]BL and [^2^H_3_]CS) versus the concentration of internal standard based on data obtained from triplicate measurements. The limits of detection (LODs) and the limits of quantification (LOQs) were calculated as the concentration of the analytes at a signal-to-noise ratio (S/N) of 3 and 10, respectively. As shown in Table 1, good linearities were obtained in the range of 0.4-500 ng/mL with correlation coefficients (R) of 0.9900-0.9995. And the LODs and LOQs were found to be in the range of 0.02-0.09 ng/mL (1.6-7.2 pg) and 0.11-0.47 ng/mL (8.8-37.6 pg), respectively.

**Table 1:** Linearity, LODs and LOQs of BRs

**Analytes**	**Linear range ng/mL (ng)**	**Regression data**			**LOD ng/mL (pg)**	**LOQ ng/mL (pg)**
		**Slope**	**Intercept**	**R value**		
28-norBL	0.4-500 (0.32-400)	0.0581	0.0081	0.9991	0.09 (7.2)	0.47 (37.6)
28-norCS	0.4-500 (0.32-400)	0.3248	−1.4261	0.9974	0.02 (1.6)	0.12 (9.6)
BL	0.4-500 (0.32-400)	0.1495	0.1126	0.9991	0.05 (4.0)	0.27 (21.6)
CS	0.4-500 (0.32-400)	0.0500	0.1434	0.9995	0.02 (1.6)	0.11 (8.8)
28-homoBL	0.4-500 (0.32-400)	0.0170	0.1574	0.9953	0.04 (3.2)	0.21 (16.8)

The reproducibility and accuracy of this method were evaluated with intra-day, inter-day measurements. The intra-day precisions were obtained with extractions of five samples over a day, and the inter-day precisions were obtained by extracting samples in continuous three days. The RSDs of inter- and intra-day precision were below 17.8%, and the relative recoveries were in the range of 79.0-117.6% (Table 2), indicating good reproducibility and accuracy of the method.

**Table 2:** **Precisions (intra- and inter-day) and recoveries of BRs in *O. sativa *seedlings (1 g FW)**

**Analytes**	**Intraday precision (RSD, %, n = 4)**			**Interday precision (RSD, %, n = 3)**			**Recovery (%, n = 4)**		
	**Low (1 ng/g)**	**Medium (5 ng/g)**	**High (25 ng/g)**	**Low (1 ng/g)**	**Medium (5 ng/g)**	**High (25 ng/g)**	**Low (1 ng/g)**	**Medium (5 ng/g)**	**High (25 ng/g)**
28-norBL	12.1	14.6	11.7	11.3	12.4	13.3	107.2	109.0	115.6
28-norCS	17.8	14.6	14.0	14.9	4.3	2.9	106.1	92.0	87.2
BL	16.7	13.3	14.8	10.2	5.0	6.1	117.6	85.3	105.2
CS	9.7	13.7	2.7	11.8	5.1	5.8	101.5	99.7	100.8
28-homoBL	11.0	9.5	2.6	14.4	4.2	14.3	79.0	87.1	97.9

### Determination of BRs in real samples

**Figure 7:**
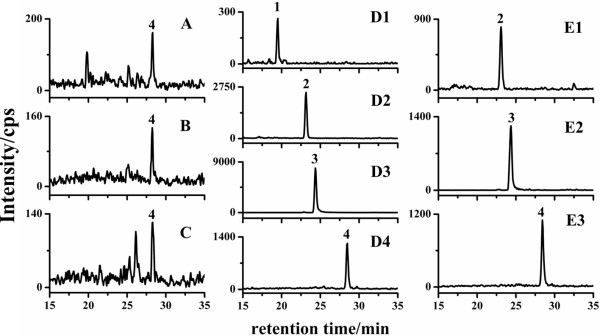
**MRM chromatographs of real samples.***O. sativa* YTA shoot (**A**), *O. sativa* YTB shoot (**B**), *B. napus* shoots (**C**), *B. napus* flower buds (**D1**-**D4**), *B. napus* flowers (**E1**-**E3**). Peaks: 1. 28-norBL; 2. 28-norCS; 3.BL; 4.CS.

The established method was applied to the determination of four endogenous BRs in five different plant tissues (*Brassica napus L.* (B. napus) leaves, *Oryza sativa ssp. indica* cv. YueTai A (YTA) (Sterile Lines) (*O. sativa* YTA) leaves, *Oryza sativa ssp. indica* cv. YueTai B(maintainer line) (*O. sativa* YTB) leaves, *B. napus* flowers and *B. napus* flower buds). As shown in Table 3, relatively high level of BRs (28-norBL, 28-norCS, BL and CS) were found in B. napus flower tissue; while quite low content of BRs (BL and CS) was found in the plant leaves (Figure 7).

**Table 3:** Amount of endogenous BRs in different plant tissues

**Analytes**	***O. sativa YTA shoot***	***O. sativa YTB shoot***	***B. napus L.*shoot**	***B. napus L. *flower bud**	***B. napus L. *flower**
BL	n.d.	n.d.	n.d.	15.84 ± 0.78	89.80 ± 7.41
CS	1.86 ± 0.11	1.33 ± 0.22	0.30 ± 0.05	13.54 ± 0.20	11.42 ± 1.54
28-norBL	n.d.	n.d.	n.d.	n.d.	6.14 ± 0.18
28-norCS	n.d.	n.d.	n.d.	5.13 ± 0.15	12.36 ± 0.56

n.d: Not detected. Unit: ng/g FW.

## Conclusion

In this study, a DL/SPE-BA/PMME-HPLC-ESI-MS/MS method for the determination of endogenous BRs in plant tissues was developed. Coupled with the previous established DL/SPE, DL/SPE-BA/PMME exhibited good purification efficiency towards BR. Compared with previously reported methods, the method proposed in this study is highly selective and solvent-saving. In addition, the endogenous BRs can be detected in 1 g (FW) leaves or 0.5 g (FW) flower tissues.

It is worth noting that the method developed here is not limited to the quantification of the above mentioned five BRs. Theoretically, all the BRs, along with their precursors and conjugates, could be analyzed using the same method because all these cis-diol-containing targets possess the similar specific affinity towards BA monoliths.

## Methods

### Reagents and materials

BR standards:28-norbrassinolide (28-norBL), 28-norcastasterone (28-norCS), brassinolide (BL), castasterone (CS), 28-homobrassinolide (28-homoBL), and stable isotope-labeled standards, [^2^H_3_]brassinolide and [^2^H_3_]castasterone, were the current commercially available BR standards, and were all purchased from Olchemim Ltd. (Olomouc, Czech Republic).

Acetonitrile and methanol with HPLC grade were purchased from Tedia Co. (Fairfield, OH, USA) and Merck (Darmstadt, Germany), respectively. Formic acid (FA, AR), NaCl, anhydrous MgSO_4_ (AR), Disodium hydrogen orthophosphate (AR), Azobisisobutyronitrile (AIBN), poly(ethylene glycol) (PEG) with molecular weights of 20,000 were bought from Sinopharm Chemical Reagent (Shanghai, China). 3-acrylamidophenylboronic acid (AAPBA) was purchased from J&K Scientific Ltd. (Beijing, China). Deionized water was obtained from a Millipore Milli-Q water purification system (Milford, MA, USA).

HiCapt GCB/PSA double layered SPE cartridge (100 mg GCB/500 mg PSA, 6 mL) was obtained from Weltech Co. (Wuhan, China). Poly(AAPBA-*co*-EDMA) boronate affinity monolith was prepared according to the previous work [27]. Briefly, 30 mg functional monomer AAPBA, 70 mg cross-linker EDMA, 265 mg methanol, 35 mg PEG 20000 and 1 wt% AIBN was homogeneously mixed in a centrifugetube and degassed by ultra-sonication. Then the prepolymerization solution was filled into fused-silica capillaries (530 mm ID) which were activated and modified with 3-(triethoxysilyl)propylmethacrylate. After sealing at both ends with silica rubber, the capillaries were polymerization at 60°C for 16 h. At last, the prepared monolith was washed with methanol with a pump.

### Plant samples

Rice (*O. sativa*, *O. sativ*a YTA and *O. sativa* YTB) shoots were harvested upon 4 months growing in the field. Three-month-old rape (*B. napus*) leaves and flowers were also harvested from the field. All the plant materials were weighted, immediately frozen in liquid nitrogen, and then stored at −80°C till analysis.

### DL/SPE-BA/PMME process for plant samples

Sample pretreatment process was shown in Figure 3. Plant tissue (1 g FW leaves, or 0.5 g FW flower tissue) was frozen in liquid nitrogen and grounded into fine powder with a mortar and pestle, and then transferred into a 10-mL centrifuge tube. Stable isotope labeled BRs [^2^H_3_]BL (2 ng) and [^2^H_3_]CS (2 ng) were added into the mixture followed by extraction with acetonitrile (5 mL/g) overnight at −20°C. The extraction, dehydration and DL/SPE were performed according to previous reported method [28]. Briefly, the acetonitrile exacted sample was centrifuged at 10,000 rpm under 4°C for 10 min. Then the supernatant was collected and the rest plant residue was re-extracted with 1 mL acetonitrile. After combining the two parts of solution, NaCl (250 mg/g FW) was added and vortexed for several minutes to induce phase separation. Anhydrous MgSO_4_ (500 mg/g FW) was added into the upper layered acetonitrile to remove residual water. After centrifugation at 10,000 rpm under 4°C for 10 min, the supernatant was collected and passed through a GCB/PSA double layered SPE cartridge (100 mg/ 500 mg, GCB/PSA) which was pre-conditioned with acetonitrile (6 mL), and the eluate was collected. The residues of BRs on the SPE cartridge were desorbed with methanol:acetonitrile (1:1, v/v, 2 mL). Then the two parts of the eluates were combined and evaporated under mild nitrogen stream followed by reconstituting in acetonitrile/buffer (1/4, v/v, pH 9.0, 1 mL). The reconstituted solution was loaded onto the previously activated boronate affinity monolith at the flow rate of 100 μL/min. After washing with acetonitrile/buffer (1/4, v/v, pH 9.0, 0.5 mL), the extracted BRs were desorbed with 3% H_2_O_2_ in acetonitrile/water (9/1, v/v, 0.6 mL) at the flow rate of 100 μL/min. The desorption solution was collected and evaporated under mild nitrogen stream followed by re-dissolving in 100 μL methanol/H_2_O ( 65/35, v/v), and 80 μL of the solution was used for the quantification of BRs by HPLC-ESI-MS/MS.

### Instrument and analytical conditions

The instrument and analytical conditions were identical to the previous work [28]. Analysis of BRs were performed on a HPLC-ESI-MS/MS system consisting of a AB SCIEX 3200 QTRAP MS/MS (Applied Biosystems, Foster City, CA, USA) with an ESI source (Turbo Ionspray), and a Shimadzu LC-20 AD HPLC system (Tokyo, Japan), which was equipped with two LC-20 AD pumps, a SIL-20A auto sampler, a CTO-20 AC column thermostat, and a DGU-20A3 degasser. The separation was achieved on a shim-pack ODS column (15 cm × 2.0 mm id, 5 μm, Shimadzu, Tokyo, Japan). The column oven temperature was set at 40°C. Binary mobile phase was used. Solvent A is 0.1% formic acid in water (v/v) and solvent B is acetonitrile. A gradient of 50 min 30–65% B, 10 min 100% B, 10 min 30% B at a flow rate of 0.2 mL/min was used. Data acquisition and processing were achieved with AB SCIEX Analyst 1.5 software.

All BRs were quantified by multiple reaction monitoring (MRM) mode in positive mode. The optimal ESI source conditions were as follows: turbo heater temperature (TEM) 400°C, ion spray voltage 5500 V, curtain gas 40 psi, nebulizing gas (gas 1) 50 psi and heated gas (gas 2) 80 psi. The collision energy (CE) and entrance potential (EP) was separately set at 20 V and 10 V. The mass transitions of BRs, optimal declustering potential (DP) and collision cell exit potential (CXP) were the same as our previously study (see supporting information) [28].

## Competing interests

The authors declare that they have no competing interests.

## Authors’ contributions

JD and YQF conceived and designed the method. JD and LJM carried out the experiments. JD, YQF and BFY wrote the manuscript. All authors read and approved the final manuscript.
